# Books and Authors

**Published:** 1908-06

**Authors:** 


### BOOKS AND AUTHORS.

---

**International Clinics.** A Quarterly of Illustrated Clinical Lectures and especially prepared articles on Treatment, Medicine, Surgery, Neurology, Pediatrics, Obstetrics, Gynecology, Orthopedics, Pathology, Dermatology, Ophthalmology, Otology, Rhinology, Laryngology, Hygiene and other topics of interest to students and practitioners. Edited by **W. T. Longcope,** M.D., Volume I., eighteenth series. Philadelphia and London: J. B. Lippincott Co. 1908. (Cloth, $2.00.)

The titles and authors in this number are the sanatorium, Lawrason Brown; treatment of syphilis by injection of soluble salts of mercury, Jean Dardal; clinical aspects of blood coagulation, Thomas R. Boggs; records of the opsonic test for diagnosis and of the employment of vaccines in infectious conditions in children, A. Dingwall Fordyce; the paratyphoid fevers, James C. Wilson; urinary acidity with reference to gastric acidity. Acid and alkaline tests in urine denied, A. L. Benedict; textural proclivities and immunity, the personal factor in medicine, Sir Dyce Duckworth; mucous colitis, David Somerville; normal temperature of the body, R. D. Rudolf; diseases of the gall bladder, John B. Deaver; deductions from a series of operations for perforated gastric and duodenal ulcer, Benjamin T. Tilton; resection of shoulder joint for suppurative disease of the bone, J. Garland Sherrill; import of digestive disturbances in the diagnosis of surgical lesions of the abdomen, Louis Frank; on fixation abscesses, Jules Thiroloix; care of the newborn, Henry Enos Tuley; perfected surgical treatment of fibroid tumors of the uterus, Lewis S. McMurtry; fracture of the spine, George L. Walton; the way of infection in tuberculosis, Lawrence F. Flick; etiology of hemoglobin uric fever, William H. Deederick.

The progress of medicine during 1907, as relates to treatment is compiled by A. A. Stevens; that relating to medicine by David L. Edsall and Verner Nisbet; and that relating to surgery, by Joseph C. Bloodgood.

The notable contributions to this number are those made by John B. Deaver, J. Garland Sherrill, Louis Frank, Lewis S. McMurtry, Henry Enos Tuley, George L. Walton, and Lawrence F. Flick. All the articles possess merit and deal with topics of considerable importance. This is one of the better volumes of recent issue.

---

**The Diagnosis and Treatment of Pulmonary Tuberculosis.** By **Francis M. Pottenger,** A.M., M.D., Medical Director of the Pottenger Sanatorium for Diseases of the Lungs and Throat, Monrovia, Cal.; Professor of Clinical Medicine in the Medical Department, University of Southern California. Octavo, pp. 377. New York: William Wood & Co. 1908. (Cloth, $3.50.)

The pursuit of the study of tuberculosis is leading constantly into new fields of investigation and as constantly demanding, or perhaps we should say creating, new literature, some of which is valuable, other some of little use. To the former class this book belongs. The examination of the patient for early tuberculosis has ever increasing importance, since this is the period when the arrest of the disease may be expected, if it is then recognised and proper precautions taken. It is in the early stage when open air treatment gives most promise as an effective measure for the arrest of disease processes. The chapter on this topic is most instructive and may be read with profit by every physician.

Prophylaxis plays an important role in this disease, and is dealt with by this author in an intelligent manner, the chapter on this topic also being both interesting and instructive. The entire volume, indeed, is filled with valuable material, well arranged, and admirably joined together. The inset marginal side headings facilitate examination of topics and subtopics, besides giving a handsome appearance to the pages. Every public sanitarian, as well as clinician, should give this work careful study.

---

**History of the Study of Medicine in the British Isles.** The Fitz-Patrick Lectures for 1905-6, delivered before the Royal College of Physicians of London. By Norman Moore, M.D., Cantab. Fellow of the Royal College of Physicians; Physician to St. Bartholomew's Hospital. Octavo, pp. 202. Oxford: Clarendon Press. 1908.

Wherever medicine is taught its history should be known. No country has contributed more to the history of medicine than Great Britain. This author, in these lectures, has added further luster to his name, and has contributed a classic to the history of medicine in the British Isles. Though he calls his book, the history of the study of medicine it is, nevertheless, a lasting tribute to the real history of medicine itself, and will be read with pleasure by every American physician of culture, who would further familiarise himself with the study of medicine as it existed in medieval times.

---

**Transactions of the American Surgical Association.** Vol. XXV. Edited by **Richard H. Harte,** M.D., Recorder of the Association.

This volume contains the papers read at the twenty-fifth annual meetings of the association, May 7, 8, and 9, 1907. There is nothing in the book to indicate the place where the meeting was held. The president, Dudley P. Allen, of Cleveland, chose for the subject of his address, "The Teaching of Surgery." A symposium on cancer of the breast, consisting of thirteen papers, constitutes a most important part of the book. Many other papers of great interest are presented, together with valuable discussions on the same, all going to make up one of the essential contributions to the literature of surgery for the year.

